# Effects of macrophages in OSCC progression

**DOI:** 10.3389/fimmu.2024.1517886

**Published:** 2025-01-14

**Authors:** Xiaodan Dong, Chunling Dong, Bo Li

**Affiliations:** ^1^ Department of Oral Anatomy and Physiology, Jilin Provincial Key Laboratory of Oral Biomedical Engineering, Hospital of Stomatology, Jilin University, Changchun, China; ^2^ School of Public Health, Jilin University, Changchun, China; ^3^ Department of Pulmonary and Critical Care Medicine, Second Hospital, Jilin University, Changchun, China

**Keywords:** oral squamous cell carcinoma, macrophages, polarization, interaction, loop

## Abstract

Macrophages are crucial immune cells within the tumor microenvironment (TME), involved in regulating tumor proliferation, invasion, metastasis, ECM remodeling, angiogenesis, and immunosuppression. Although more and more experimental evidence and clinical data indicate that macrophages are involved in the onset and progression of oral squamous cell carcinoma (OSCC), the exact pathogenesis of OSCC associated with macrophages has not been fully elucidated. Enhanced knowledge of the molecular mechanisms involving macrophages in OSCC will aid in the creation of treatments targeted specifically at macrophages. This review outlines the pro-tumoral and anti-tumoral effects of macrophages in OSCC, emphasizing the interaction between OSCC cells and macrophages. It can provide theoretical basis for the establishment of complex regulatory network centered on macrophages and explore novel therapeutic strategies for OSCC.

## 1 Introduction

Oral squamous cell carcinoma (OSCC) ranks as the sixth most prevalent cancer globally, with over 90% of oral malignancies attributed to its occurrence (1–3). The characteristics of OSCC include rapid proliferation, high local infiltration rate, strong metastasis ability and poor prognosis (4–6). Due to inadequate knowledge of the pathogenesis of OSCC, the survival rate after five years is below 50%. Recent research indicates that ETS1, OTUB1 and PTP4A1 could promote OSCC proliferation, invasion, and metastasis (7–9). Gong et al. discovered that genes related to ECM, especially THBS1, can impact OSCC biological characteristics, immunotherapeutic responses and prognosis (10). In OSCC, the spatial distribution of vessel density in the tumor center (sparse vessels) is different from that in the invasion front (high vessel density), revealing the microvascular spatial heterogeneity (11).

**Figure 1:**
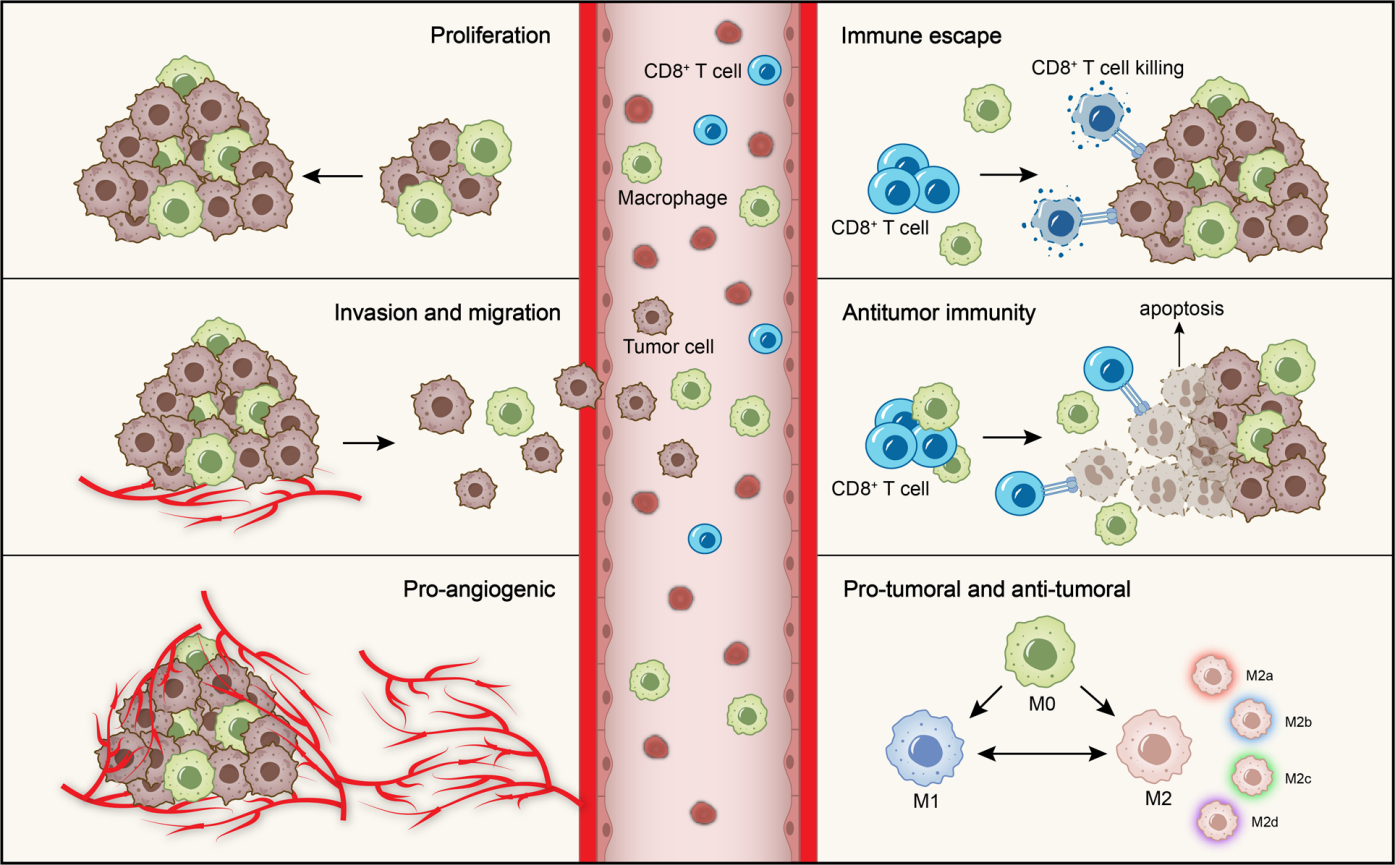
Role of macrophages in OSCC. The role of macrophages in OSCC is mainly divided into the pro-tumoral and anti-tumoral effects. The pro-tumoral effects include promoting tumor cells proliferation, invasion and migration, angiogenesis and immune escape. The anti-tumoral effect is mainly manifested in anti-tumor immunity. Moreover, macrophages are polarized into M1 and M2 phenotypes with anti-tumoral and pro-tumoral effects. M2 macrophages further subdivided into four subtypes according to different microenvironmental stimuli.

Scientists have begun to focus on the role of macrophages during oral oncogenesis as well as the prognostic significance of macrophages in OSCC survival and response to standard treatment regimens, and have discussed new concepts for the macrophages as immunotherapeutic targets of OSCC and therapeutic strategies for TAMs (12, 13). Although more and more experimental evidence and clinical data indicate that macrophages are involved in the onset and progression of OSCC (14–18), the exact pathogenesis of OSCC associated with macrophages has not been fully elucidated. Enhanced knowledge of the molecular mechanisms involving macrophages in OSCC will aid in the creation of treatments targeted specifically at macrophages. This review outlines the pro-tumoral and anti-tumoral effects of macrophages in OSCC (**Figure 1**), emphasizing the interaction between OSCC cells and macrophages. It can provide theoretical basis for the establishment of complex regulatory network centered on macrophages and explore novel therapeutic strategies for OSCC.

## 2 Macrophages

### 2.1 Function and polarization of macrophages

Macrophages exhibit a significant impact on the development of different illnesses, including cancers (19–27) through their phagocytosis (27, 28), antigen presentation (29), immunomodulatory (29) and secretory functions (30–32). Macrophages are polarized into M1 and M2 phenotypes with the different surface markers and functions. M2 macrophages include four different subtypes: M2a, M2b, M2c, and M2d.

### 2.2 Effects of tumor-associated macrophages

**Figure 2:**
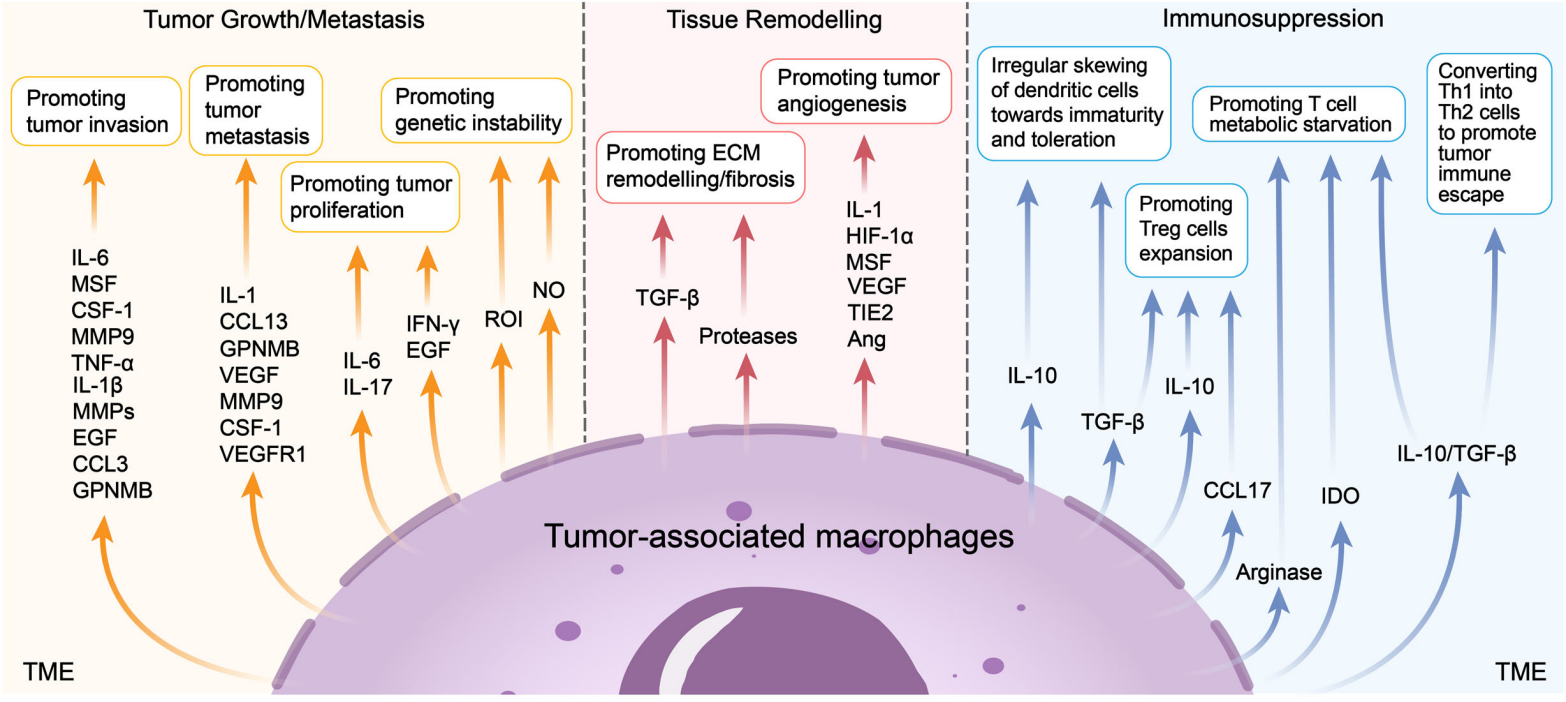
Pro-tumoral functions of TAMs. TAMs could promote tumor growth or metastasis, tissue remodelling and immunosuppression. Recent studies show that TAMs produce mediators such as IL-6, MSF et al. to promote tumor invasion, produce IL-1, CCL13 et al. to promote tumor metastasis, produce IL-6, IL-17 et al. to promote tumor proliferation, produce ROI and NO to promote tumor genetic instability. TAMs also produce TGF-β and proteases to promote ECM remodelling/fibrosis, produce IL-1, HIF-1α et al. to promote tumor angiogenesis. TAMs play a critical role of immunosuppression in the TME, such as promoting the irregular skewing of dendritic cells towards immaturity and toleration, promoting Treg cells expansion, T cell metabolic starvation and converting Th1 into Th2 cells to promote tumor immune escape.

Tumor-associated macrophages (TAMs) are crucial immune cells within the tumor microenvironment (TME), involved in regulating tumor proliferation, invasion, metastasis, ECM remodeling, angiogenesis, and immunosuppression (**Figure 2**). The interactions and crosstalk between tumor cells and macrophages play a crucial role in tumor progression. The pro-tumoral and anti-tumoral effects of TAMs are closely related to their infiltration (33, 34), polarization (34–37) and reprogramming (38–41). M1 TAMs are generally thought to exert anti-tumoral effects, while M2 TAMs present pro-tumoral effects. Recent research indicates that M1 TAMs may also contribute to tumor progression, including OSCC (15, 42–44). The distinct expression pattern of CXCL9:SPP1 in TME controls TAMs polarity and is closely related to immune cell profile, anti-tumoral factors, and patient prognosis (45).

### 2.3 Polarization of tumor-associated macrophages

Relevant studies have shown that OSCC cells-derived mediators promote macrophages M1 and M2 polarization through different signaling pathways. Zhang and Le et al. found that eIF5Ahpu in OSCC cells can promote M2 TAMs polarization (46). Ai et al. claimed that OSCC cells-derived Circ-ILF2 promoted M2 macrophages polarization, providing the novel insights into immunotherapy (47). Cong et al. pointed out that SOAT1 regulated by ETS1 induces M2 TAMs polarization (48). Hsieh et al. offered that HDAC6 in OSCC cells enhance M2 macrophages polarization through AP-1/IL-13 (49). Zhang et al. demonstrated that HMGB1 in OSCC cells and macrophages can promote M2 macrophages polarization via OSCC cells paracrine and macrophages autocrine IL-10/TGF-β respectively (50). Meanwhile, OSCC cells paracrine and macrophages endogenous HMGB1 can promote M1 macrophages polarization via TLR4/NF-κB signaling pathway (50). In addition, Chen et al. revealed that exosome-transferred THBS1 derived from tumor cells polarized macrophages to the M1 phenotype in OSCC (51). The above studies indicated that OSCC cells-derived mediators mainly promote M2 macrophages polarization. The potential research direction can be placed on the regulation of M1 and M2 polarization by macrophages endogenous mediators, and the reprogramming of macrophages polarization.

## 3 Interaction between OSCC cells and macrophages

### 3.1 TAMs-mediated interaction in OSCC

**Figure 3:**
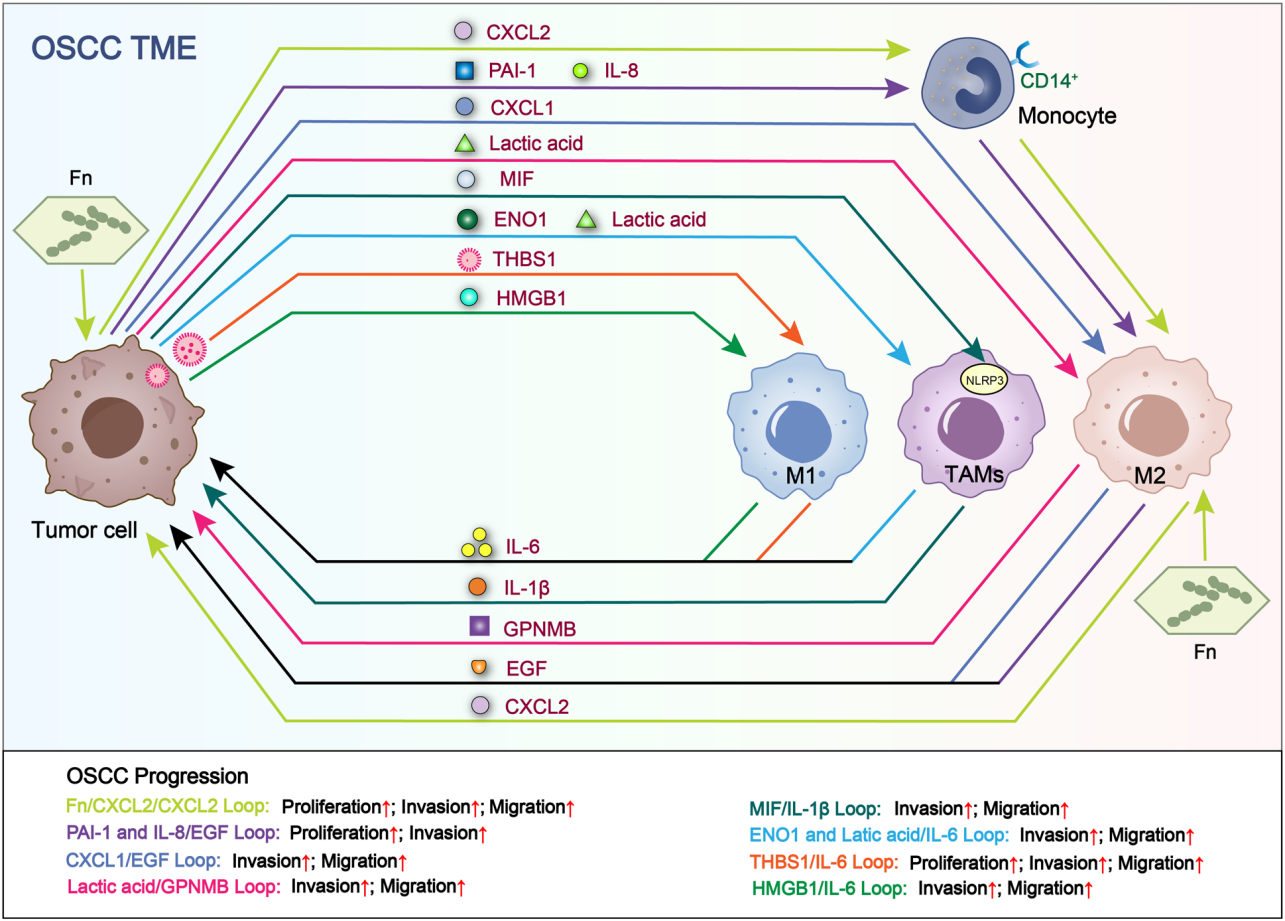
Loops related to interactions between tumor cells and macrophages. Tumor cell-derived RNAs, proteins and metabolites increase the secretion of cytokines in TAMs. In turn, the above TAM-derived cytokines promote OSCC proliferation, invasion and migration. Fn/CXCL2/CXCL2 loop and THBS1/IL-6 loop facilitate the proliferation, migration and invasion of OSCC. PAI-1 and IL-8/EGF loop facilitate the proliferation and invasion of OSCC. CXCL1/EGF loop, Lactic acid/GPNMB loop, MIF/IL-1β loop, ENO1 and Latic acid/IL-6 loop and HMGB1/IL-6 loop facilitate the invasion and migration of OSCC.

Choi et al. reported that MIF/NLRP3/IL-1β loop between cancer cells and TAMs promotes OSCC invasion and migration (**Figure 3**; **Table 1**) (17). Li et al. found that alpha-enolase (ENO1) and lactic acid derived from tumor cells stimulates OSCC invasion and migration by coordinating IL-6 release from TAMs (**Figure 3**; **Table 1**) (52).

**Table 1:** Pro-tumoral and anti-tumoral role of macrophages in OSCC.

Role	Effect	Critical factors or Mechanism	Reference
Pro-tumoral	Promote proliferation	**IL-6** derived from M1 macrophages	(15)
		**miRNA-23a-3p** derived from M2 macrophages	(71)
		**EGF** derived from M2 TAMs (CD206^+^)	(56)
		M2 polarization induced by **GABA**	(75)
		**CXCL2** derived from M1 macrophages	(57)
	Promote invasion and migration	**HMGB1** derived from macrophages	(50)
		**IL-1β** derived from TAMs	(17)
		**IL-6** derived from TAMs and M1, M2 macrophages	(52, 42, 15, 72)
		**GPNMB** and **miRNA-23a-3p** derived from M2 macrophages	(53, 71)
		**EGF** and **CCL13** derived from M2 TAMs	(56, 73)
		M2 polarization induced by lactic acid	(53)
		**CXCL2** derived from M1 macrophages	(57)
	Promote angiogenesis	Macrophages reprogramming towards a pro-angiogenic phenotype induced by **TGF-β^+^ small extracellular vesicles**	(63)
	Promote immune escape of tumor cells	PD-L1 expression in TAMs through JAK2/STAT3 signaling pathway induced by **GM-CSF**	(78)
		PD-L1 expression in macrophages through IL-6/STAT3 signaling pathway induced by **HMGB1**	(50)
	Related to lymph node relapse	**CCL22** derived from M2 TAMs	(85)
	Promote tumor progression	M1 polarization induced by **HMGB1**	(42, 50)
Anti-tumoral	Promote anti-tumor immunity	Lymph nodes CD169^+^ macrophages activation induced by **Naringenin**	(87)
		M1 polarization induced by the combination of **anti-PD-L1 and regorafenib**	(95)
	Inhibit tumor progression	TAMs reprogramming towards an antitumor phenotype treated by **Curcumin**	(94)

Bold texts highlight critical factors.

### 3.2 M2 TAMs-mediated interaction in OSCC

**Figure 4:**
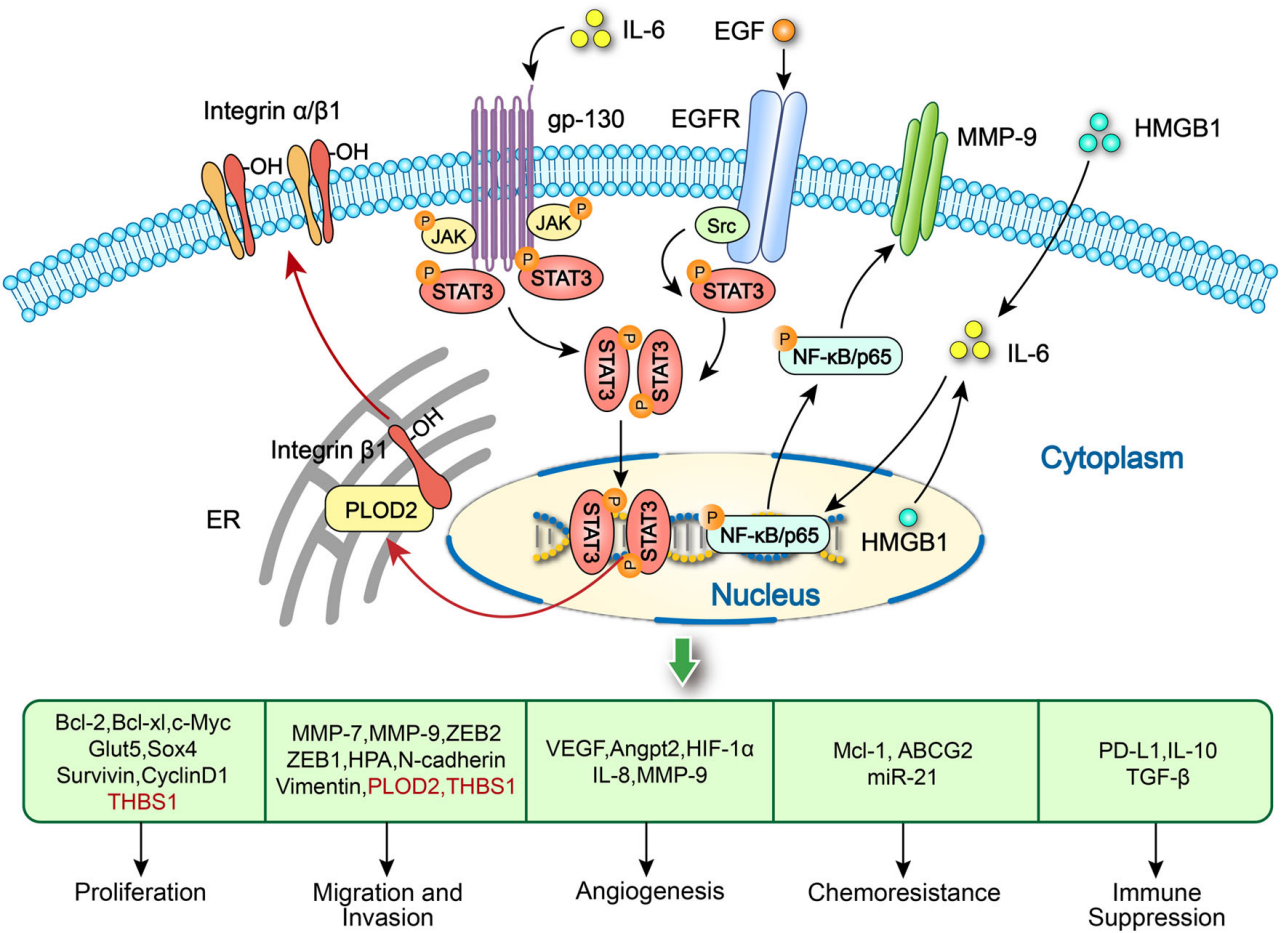
Activation of STAT3 and NF-κB/p65 signaling pathways in tumor cells promote OSCC progression. PLOD2-driven IL-6/STAT3 signaling promotes the invasion and metastasis of OSCC via activation of integrin β1. IL-6 released by M1-like TAMs activates the STAT3 signaling to produce exosome-THBS1 in OSCC cells, promoting tumor proliferation, invasion and migration. EGF produced by CD206^+^ TAMs increase the progression of OSCC by activating the STAT3 pathway. HMGB1 in both macrophages and OSCC cells might regulate the IL-6/NF-κB pathway to increase MMP-9 expression and further promote tumor growth and invasion.

Li et al. revealed that lactic acid promotes macrophages M2 polarization and the release of glycoprotein non-transfer protein B (GPNMB), facilitating OSCC EMT, invasion and migration through CD44 signaling pathway (**Figure 3**; **Table 1**) (53). Li et al. also observed that OSCC cell-derived CXCL1 promotes macrophages M2 polarization and release epidermal growth factor (EGF) (54). Then, macrophage-derived EGF facilitates OSCC invasion and migration through NF-κB signaling. Moriyama et al. reported that OSCC cells induce the differentiation of CD14^+^ monocytes into CD206^+^ TAMs by secreting plasminogen IL-8 and PAI-1 (55). Subsequently, CD206^+^ TAMs release EGF to promote OSCC proliferation and invasion (**Figures 3**, **4**; **Table 1**) (56). However, no evidence indicates the association between OSCC cells-derived IL-8/PAI-1 and EGF release in macrophages. Yang et al. demonstrated that CXCL2 derived from OSCC cells stimulated by Fusobacterium nucleatum (Fn) promotes the recruitment and M2 polarization of macrophages (57). M2 macrophages stimulated by Fn release CXCL2 to promote OSCC proliferation, invasion and migration (**Figure 3**; **Table 1**) (57). Fn induces the production and release of CXCL2 from OSCC cells and macrophages via NF-κB pathway, and enhances the CXCL2-mediated interaction between OSCC cells and macrophages, highlighting the amplification effect of Fn on the positive feedback loop between them.

### 3.3 M1 TAMs-mediated interaction in OSCC

**Figure 5:**
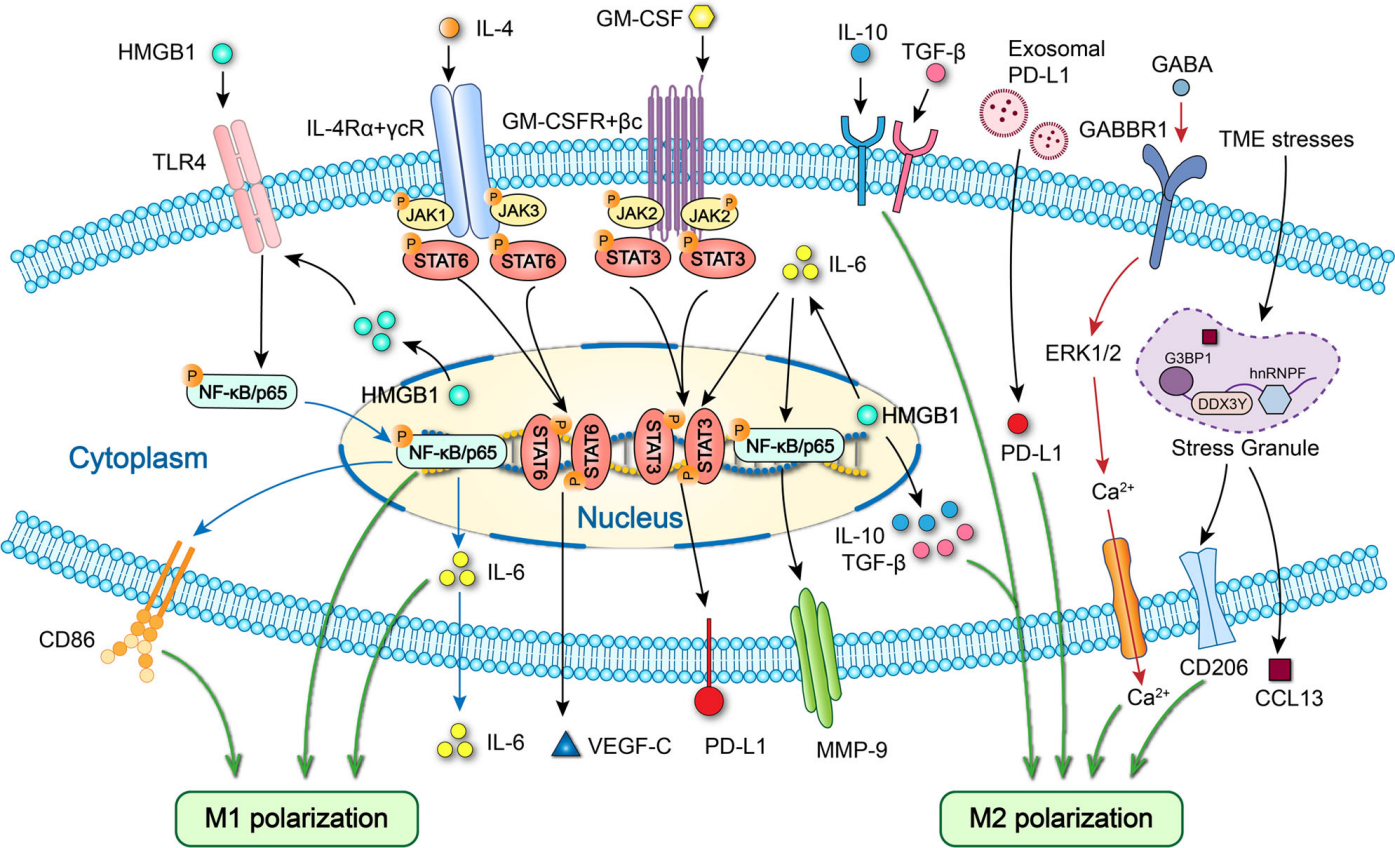
Specific cell signaling related to polarization and cytokine expression of macrophages in OSCC. HMGB1 promotes M1 polarization and IL-6 secretion via TLR4/NF-κB pathway and M2 polarization via IL-10/TGF-β. Endoplasmic reticulum stress promotes the release of exosomal PD-L1 and upregulates PD-L1 expression in macrophages to drive M2 macrophages polarization. GABA promotes M2 macrophages polarization by activating GABBR1/ERK/Ca^2+^. Elevated stress granule formation in stressed M2 TAMs enhances the expression of CCL13 by improving DDX3Y/hnRNPF-mediated CCL13 mRNA stability. HMGB1 increases MMP-9 expression via IL-6/NF-κB pathway and PD-L1 expression via IL-6/STAT3 signaling. GM-CSF upregulates PD-L1 expression via JAK2/STAT3 signaling. IL-4 increases VEGF-C secretion via STAT6 signaling.

Recent studies have shown that M1 TAMs promotes OSCC progression by interacting with tumor cells to form a positive feedback loop. Chen et al. claimed that exosome-transferred THBS1 derived from tumor cells polarized macrophages to the M1 phenotype in OSCC (51). Wang et al. further elucidated that M1 TAMs promotes the colony forming, invasion, migration, microsphere and xenograft forming abilities of OSCC cells through the IL-6/STAT3/THBS1 feedback loop (**Figures 3**, **4**; **Table 1**) (15). The above two studies can form a complete THBS1/IL-6 loop to promote the proliferation, invasion and migration of OSCC. Li et al. found that HMGB1 derived from tumor cells induces M1 polarization of macrophages and its IL-6 secretion through the NF-κB pathway, enhancing OSCC invasion and migration (**Figures 3**, **5**; **Table 1**) (42).

Macrophages, like spiders, use RNA, proteins, cytokines and metabolites to build complex networks that dynamically monitor and regulate the TME and participate in OSCC progression through intercellular interactions and crosstalk (15, 42, 52, 53, 58–60). It is worth noting that TAMs-derived IL-6 actively participates in the interaction between tumor cells and macrophages and dramatically enhances the proliferation, invasion and migration of OSCC, which deserves further research and is expected to become a potential therapeutic target. Meanwhile, the regulation of TAMs by lactic acid and its role in the interaction between tumor cells and macrophages have attracted the attention of researchers.

## 4 Pro-tumoral effects of macrophages in OSCC progression

### 4.1 Macrophages promote OSCC proliferation, invasion and migration

#### 4.1.1 TAMs promote OSCC proliferation, invasion and migration

Zhang et al. proved that HMGB1 derived from macrophages enhanced OSCC proliferation, invasion and migration by forming the immunosuppressive TME via the IL-6/STAT3/PD-L1 and IL-6/NF-κB/MMP-9 pathways (**Figures 4**, **5**; **Table 1**) (50). Silva et al. observed that macrophages activation and polarization induced by overexpression of TWIST1 and CSF1 in OSCC cells facilitate OSCC invasion (61). Lamers et al. claimed that macrophages-derived IL-6 promotes the migration ability of OSCC cells (62). Whiteside et al. found that TAMs treated with TGF-β^+^ small extracellular vesicles derived from tumor cells promote HNSCC proliferation and migration (**Table 1**) (63). Wang et al. revealed that OSCC cells fused with macrophages enhances their migration ability by activating the CCL22/CCR4 axis (64).

#### 4.1.2 M2 TAMs promote OSCC proliferation, invasion and migration

It is well-known that M2 TAMs are a facilitator of OSCC proliferation, invasion and migration through their pro-tumoral effects. Zhuo and Zhao et al. pointed out that M2 TAMs infiltration induced by SPP1 overexpression in tumor cells may enhance lymph node metastasis in HNC (65). Chen et al. offered that M2 TAMs induced by Fn-mediated OSCC cells-derived lactate adsorption promote OSCC invasion (14). Liu and Wu et al. claimed that M2 TAMs, induced by OSCC-CSC-sEVs through transferring UCA1 and targeting LAMC2 in macrophages, enhance OSCC migration and invasion *in vitro* and the tumorigenicity of xenografts *in vivo* (66). Liu et al. found that TTYH3 expression is upregulated in OSCC cells and M2 TAMs, and TTYH3 silence in OSCC cells and macrophages could inhibit OSCC proliferation, migration, and invasion through inhibiting M2 TAMs polarization (67). Kirkwood et al. revealed that Dusp1 gene deficiency in macrophages increases macrophages infiltration and enhances M2 macrophages polarization, promoting OSCC growth and migration (68). Liu et al. demonstrated that macrophages CCR7 facilitates M2 macrophages polarization, enhancing OSCC proliferation, invasion and migration (69). The above two studies by a research group highlight that macrophages CCR7 facilitates OSCC progression via Dusp1-regulated M2 macrophages polarization (68, 69). In terms of mechanism, M2 TAMs polarization is dependent on the LAMC2, TTYH3 and CCR7 expression, and Dusp1 absence. These studies suggest macrophages LAMC2, TTYH3, Dusp1, CCR7 and Dusp1-encoded MKP-1 are potential targets to modulate macrophages to inhibit OSCC progression.

M2 macrophages promote OSCC proliferation, invasion and migration by releasing microRNA, cytokines and chemokines. Dai and Zhang et al. observed that exosomal miR31-5p derived from M2 macrophages promotes OSCC proliferation and tumorigenesis through inhibiting tumor suppressor LATS2 gene via suppressing the Hippo signaling pathway (70). Li et al. demonstrated that the M2 TAMs-derived exosomal miRNA-23a-3p accelerates OSCC proliferation and invasion through PTEN targeting (**Table 1**) (71). Saito et al. noted that IL−6 secreted by M2 TAMs facilitates OSCC invasion and metastasis enhanced by PLOD2−integrin β1 axis (**Figure 4**; **Table 1**) (72). Wang et al. found that M2 TAMs enhance OSCC migration and metastasis through stress granule-regulated CCL13 release (**Figure 5**; **Table 1**) (73).

#### 4.1.3 M1 TAMs promote OSCC proliferation, invasion and migration

The role of M1 TAMs in OSCC is highly controversial due to their emerging pro-tumoral effects. Yang et al. claimed that M1 TAMs promote OSCC proliferation, invasion and migration through the activation of GDF15-mediated ErbB2 phosphorylation (43). Although there are few studies involved in this field, the pro-tumoral effects of M1 TAMs may be gradually revealed with the application and popularization of new technologies such as single-cell sequencing and organoid modeling. The pro-tumoral effects of M1 TAMs on OSCC proliferation, invasion and migration may be focused on oxidative stress, pro-inflammatory environment or the release of inflammatory cytokines such as IL-6.

### 4.2 Macrophages promote OSCC immunosuppression and immune escape

#### 4.2.1 M2 macrophages recruitment and polarization

M2 macrophages recruitment and polarization are known to create the immunosuppressive microenvironments in OSCC. Rangel et al. reported that the recruitment of M2 macrophages within the tumor induced by TP53 gain-of-function mutation of OSCC cells promotes the immunosuppression and immune escape (74). Tang et al. found that M2 macrophages polarization induced by OSCC cells-derived GABA through GABBR1/ERK/Ca2^2+^ activation promotes immunosuppression (**Figure 5**; **Table 1**) (75). Wu and Yuan et al. noted that M2 macrophages polarization promoted by the exosomal PD-L1 derived from endoplasmic reticulum stressed OSCC cells causes immunosuppression (**Figure 5**) (76). Liu and Wu et al. observed that M2 macrophages polarization induced by cancer stem cell-derived UCA1 carried by small extracellular vesicles via a LAMC2-mediated PI3K/AKT axis facilitates immunosuppression by suppressing CD4^+^ T-cell proliferation and IFN-γ production (66). Inhibition of M2 macrophages polarization can be used as a long-term therapeutic strategy to control or reverse immunosuppression and immune escape. The new targets should be macrophages surface receptors that mediate signaling pathways associated with M2 macrophages polarization, including CCR7 and TLR4.

#### 4.2.2 Macrophages protein expression

Several studies proved that macrophages protein expression can promote OSCC immunosuppression and immune escape. Tao et al. offered that the infiltration of macrophages might lead to PD-L1-mediated immunosuppression and immune escape in OSCC (77). Zhou et al. demonstrated that the increased PD-L1 expression in TAMs induced by OSCC cells-derived GM-CSF via the JAK2/STAT3 pathway promotes immune evasion (**Figure 5**; **Table 1**) (78). Zhang et al. offered that HMGB1 in macrophages promotes immunosuppression and immune escape via the IL-6/STAT3/PD-L1 pathway (**Figure 5**; **Table 1**) (50). Ekalaksananan et al. revealed that IDO expression in macrophages induced by the exosomal EBER-1 via RIG-I/IL-6/TNF-α pathway facilitates immunosuppression in OSCC through repressing T-cell function (79). Ding et al. demonstrated that upregulated PLIN2 in CD68^+^ TAMs contributes to a high PLIN2 expression in the microenvironment, inducing immunosuppression in OSCC (80). Follow-up studies should verify whether proteins expressed by macrophages, including PD-L1, HMGB1, IDO and PLIN2, can be used as potential therapeutic directions for OSCC.

### 4.3 Macrophages promote OSCC angiogenesis

It is well known that macrophages play an important role in angiogenesis because they can produce pro-angiogenic factors. Whiteside et al. found that adenosine carried by tumor-derived exosomes induced A_2B_R-mediated M2 polarization of macrophages and increased their secretion of angiogenic factors, resulting in promoting angiogenesis (81). Subsequently, they demonstrated that TGF-β carried by tumor-derived small extracellular vesicles can induce the pro-angiogenic phenotype of macrophages in HNSCC, characterized by the increased pro-angiogenic factors and enhanced pro-angiogenic functions (**Table 1**) (63). However, the detailed signaling pathways related to pro-angiogenic factors in macrophages and tumor cells and targeted drug interventions need to be further investigated.

### 4.4 Other pro-tumoral effects of macrophages in OSCC

Qin et al. pointed out that TAMs-induced pro-tumoral cancer-related inflammation can be inhibited by ALDH3A1 overexpression in tumor cells, thereby reducing the OSCC tumorigenesis (82). Fazioli et al. noted that high expression of both CD163^+^ and CD11c^+^ macrophages in inflammation area is positively associated with poorly differentiated (grade 3, G3) lesions in OTSCC patients (83). Gong and Li et al. reported that the increased DOK3 expression in TAMs induced by Porphyromonas gingivalis facilitates OSCC recurrence, possibly through TNF and MAPK signaling pathways (84). Kimura et al. demonstrated that TAMs CCL22 expression facilitates lymphangiogenesis and cause lymph node relapse via VEGF-C expression within the TME and the IL-4/STAT6 signaling pathway in early stage tongue SCC (**Figure 5**; **Table 1**) (85). Miguel et al. offered that more IL-17^+^ macrophages exist in the highly malignant OTSCC, suggesting their pro-tumoral role (86). OSCC-related inflammation mediated by TAMs and gene expression and cytokine secretion of TAMs exhibit pro-tumoral effects of macrophages and are closely related to OSCC recurrence.

## 5 Anti-tumoral effect of macrophages in OSCC

Recently, some studies have investigated the anti-tumoral effect of macrophages in OSCC. Kawahara and Nakayama et al. proved that lymph node CD169^+^ macrophages participate in antitumor immunity mediated by T cells (**Table 1**) (87). Yang et al. claimed that M1 macrophages polarization promoted by tumor cells B7H4 silence via PD-1/STAT3 signaling inhibits the OSCC progression (88). Yang et al. observed that RGS12 in macrophages suppresses OSCC by promoting M1 TAMs polarization via MYCBP2/KIF2A signaling pathways (89). Li and Zou et al. found that exosomal LBX1-AS1 secreted from macrophages with RBPJ overexpression suppresses the OSCC development via miR-182-5p/FOXO3 (90).

So far, the relevant studies on the anti-tumoral effect of macrophages in OSCC have stuck to clarifying the detailed mechanism of macrophages M1 polarization. To investigate the function and mechanism of CD169^+^ macrophages and further explore the anti-tumoral mechanism of macrophages is of great significance for developing new OSCC treatment methods and improving the therapeutic effect. Targeted activation of CD169^+^ macrophages and upregulation of RGS12 and RBPJ expression in macrophages may provide new strategies for OSCC therapy and contribute to the development of new anti-OSCC drugs.

## 6 Macrophage-associated OSCC treatment

Considering the important role of macrophages in OSCC progression, targeting macrophages to interfere with OSCC progression has become a new and promising therapeutic strategy. Yu and Zhao et al. observed that M2 TAMs-induced immunosuppression is reversed by HSA-coated perfluorocarbon carrying oxygen via targeting HIF-1α, inhibiting the OSCC growth (**Table 2**) (91). Priyadharsini et al. pointed out that engineered exoASO-STAT6 may be an effective monotherapy for OSCC (**Table 2**) (92). Zhu and Yang et al. found that macrophages membrane-encapsulated drug-carrying nanoparticles is a potential targeted therapy strategy for OSCC due to the tumor targeting and immune escape ability of macrophages (**Table 2**) (93). Targeted therapeutic strategies using drug-carrying nanoparticles and engineering techniques have become an effective means to treat OSCC. The next research focus is to improve the tumor targeting and immune escape ability of engineered exosomes and drug-carrying nanoparticles, as well as the therapeutic efficiency.

**Table 2:** Macrophage-associated treatment, prognosis and biomarker in OSCC.

Progression	Effect	Mechanism or Critical factors	Reference
Treatment	Inhibit OSCC growth	M2 TAMs-induced immunosuppression is reversed by **HSA-coated perfluorocarbon carrying oxygen** via targeting HIF-1α	(15)
	Inhibit OSCC tumor growth	**exoASO-STAT6** selectively silenced STAT6 expression in TAMs, converting M2 TAMs to M1 phenotype	(92)
	A potential targeted therapy strategy for OSCC	**Macrophages membrane-encapsulated drug-carrying nanoparticles** due to tumor targeting and immune escape ability of macrophages	(93)
	Inhibit OSCC invasion and migration	**Melatonin** disrupts MIF/NLRP3/IL-1β loop between cancer cells and TAMs	(17)
	Fight against OSCC	**Naringenin** activates lymph node CD169^+^ macrophages and enhances anti-tumoral immunity	(87)
	Suppress migration and invasion of OSCC	**Curcumin** can reprogram TAMs from a pro-tumoral phenotype towards an anti-tumoral phenotype by suppressing MAO-A/STAT6 signaling pathway	(94)
	Enhance antitumor immune efficacy of anti-PD-L1 immunotherapy on OSCC	**Regorafenib** promotes M1 macrophages polarization	(95)
Prognosis	Mean reduced survival and poor prognosis for OSCC patients	A large number of TAMs, especially **M2 TAMs**	(96)
	Predict 20-year non-disease-specific and 5-year disease-specific survival in OPSCC patients	Level of **CD68^+^ macrophages** infiltration	(97, 98)
	Have no any prognostic significance in OSCC	**CD68^+^ TAMs**	(99)
	Associated with better disease-free survival in high-grade OTSCC	High expression of **CD11c^+^ macrophages** in inflammation area	(83)
	Correlated with poor overall and disease-free survival in OSCC and OTSCC, suggesting adverse prognosis	**CD163^+^ TAMs**	(83, 99, 100)
	Predict unfavorable prognosis in Porphyromonas gingivalis-infected OSCC	**M2 TAMs** infiltration	(101)
	Cause worst overall survival of HPV16-related OPSCC	**TREM-1^+^ macrophages**	(102)
	Related to advanced stages and (reduced survival rates) poor prognosis of OSCC	High **TREM-2** expression in **TAMs**	(103)
	Associated with advanced stages and decreased progression-free survival of OSCC	High **IDO1** expression in **TAMs**	(104)
	Have poor survival	Upregulated **PLIN2** in **CD68^+^ TAMs**	(80)
Biomarker	A dependable marker for evaluating aggressiveness of OSCC	**TAMs**	(105)
	A potential candidate biomarker for recurrence and/or metastasis (R/M) in early stage of OTSCC	**M2 macrophages abundance**	(106)
	A biomarker for OSCC combination therapy including immunotherapy	**CD68^+^ and PD-L1^+^ macrophages**	(107)
	A prognostic biomarker for OSCC	Lymph node **CD169^+^ macrophages**	(87)
	A diagnostic biomarker for OSCC therapy	TAMs-derived **LBX1-AS1**	(90)
	An essential OSCC biomarker	**RGS12** in macrophages	(89)
	A prognostic biomarker for OSCC	**TREM-2** in TAMs	(103)
	A suitable biomarker for OSCC metastasis	**IDO1** activity of TAMs	(104)

Bold texts highlight critical factors.

Choi et al. reported that melatonin inhibits OSCC invasion and migration by disrupting MIF/NLRP3/IL-1β loop between cancer cells and TAMs (**Table 2**) (17). Kawahara and Nakayama et al. claimed that naringenin fights against OSCC through activating lymph node CD169^+^ macrophages and enhancing anti-tumoral immunity, which is a promising drug (**Tables 1**, **2**) (87). Li et al. pointed out that Curcumin can reprogram TAMs from a pro-tumoral phenotype towards an anti-tumoral phenotype by suppressing the MAO-A/STAT6 signaling pathway (**Tables 1**, **2**) (94). Hsu and Tu et al. demonstrated that regorafenib enhances anti-OSCC efficacy of anti-PD-L1 by promoting M1 macrophages polarization (**Tables 1**, **2**) (95). Disrupting pro-tumoral interactive loops between OSCC cells and TAMs, activating or reprogramming TAMs toward a proinflammatory or anti-tumoral phenotype using traditional Chinese medicine and common medicine are good options for OSCC treatment. The next research direction is to screen the effective drug ingredients and clarify the specific treatment mechanism, and improve the efficiency of drug therapy by using new technologies and drug delivery system.

## 7 Macrophage-associated OSCC prognosis

The intervention targeting macrophage can significantly improve the prognosis of OSCC patients, and the macrophage-related prognostic value is worthy of evaluation. Ferreira et al. offered that a large number of TAMs, especially M2 TAMs, mean reduced survival and poor prognosis for OSCC patients (**Table 2**) (96). Aarstad et al. proved that level of CD68^+^ macrophages infiltration predicts 20-year non-disease-specific and 5-year disease-specific survival in OPSCC patients (**Table 2**) (97, 98). However, Foey et al. reported that CD68^+^ TAMs do not have any prognostic significance in OSCC (**Table 2**) (99). Fazioli et al. proved that high expression of CD11c^+^ macrophages in inflammation area is associated with better disease-free survival in high-grade OTSCC (**Table 2**) (83). Several studies have shown that CD163^+^ TAMs are correlated with poor overall and disease-free survival in OSCC and OTSCC, suggesting the adverse prognosis (**Table 2**) (83, 99, 100). Gong and Li et al. noted that the increased levels of DOK3 expression and M2 TAMs infiltration predict the unfavorable prognosis in Porphyromonas gingivalis-infected OSCC (**Table 2**) (101). The above studies show that the association between CD68^+^ macrophages and OSCC prognosis remains controversial and needs further investigation. There is no doubt that the infiltration level of M2 TAMs is closely related to the poor prognosis of OSCC. Obviously, the intervention targeting M2 TAMs infiltration can significantly improve the prognosis. The key to further research is how to inhibit the M2 polarization of TAMs.

Azzimonti et al. pointed out that TREM-1^+^ macrophages play a very important role in the progression of HPV16-related OPSCC, and TREM-1 positivity causes the worst overall survival (**Table 2**) (102). Struckmeier et al. observed that high TREM-2 expression in TAMs was related to advanced stages and (reduced survival rates) poor prognosis of OSCC (**Table 2**) (103). Struckmeier et al. also claimed that high IDO1 expression in TAMs was associated with advanced stages and decreased progression-free survival of OSCC (**Table 2**) (104). Ding et al. offered that patients with upregulated PLIN2 in CD68^+^ TAMs are prone to metastasis after surgery and have poor survival (**Table 2**) (80). These studies contribute to further elucidating the specific role and detailed mechanisms of macrophages in the prognosis of OSCC, thus providing new targets for OSCC treatment. Further screening of key TAMs proteins closely related to tumor development and prognosis, and targeted intervention of these proteins, including TREM-1/2, IDO1 and PLIN2, are expected to control the progression of OSCC and improve its survival.

## 8 Macrophage-associated biomarker in OSCC

Since macrophages can interfere with OSCC progression from multiple aspects, such as proliferation, invasion, migration, angiogenesis, and immune escape, macrophage-related biomarkers are worthy of attention. Mukherjee et al. pointed out that TAMs are a dependable marker for evaluating the aggressiveness of OSCC (**Table 2**) (105). Azuma et al. claimed that the M2 macrophages abundance is a potential candidate biomarker for recurrence and/or metastasis (R/M) in the early stage of OTSCC (**Table 2**) (106). Zhang et al. offered that CD68^+^ and PD-L1^+^ macrophages may serve as a biomarker for OSCC combination therapy including immunotherapy (**Table 2**) (107). Kawahara and Nakayama et al. found that lymph node CD169^+^ macrophages participate in anti-tumoral immunity mediated by T cells and can be a prognostic biomarker for OSCC (**Table 1**) (**Table 2**) (87). These studies indicate that TAMs surface markers, including CD68\CD163\CD169\CD206 and PD-L1, can be used as biomarkers to evaluate the invasion, metastasis, treatment recurrence and prognosis of OSCC.

Li and Zou et al. reported that TAMs-derived LBX1-AS1 is probably a diagnostic biomarker for OSCC therapy (**Table 2**) (90). Yang et al. revealed that RGS12 in macrophages is an essential OSCC biomarker (**Table 2**) (89). Struckmeier et al. proved that TREM-2 in TAMs can be a prognostic biomarker for OSCC (**Table 2**) (103). Struckmeier et al. also demonstrated that IDO1 activity of TAMs could be a suitable biomarker for OSCC metastasis (**Table 2**) (104). TAMs-derived lncRNA and proteins could be biomarkers of OSCC progression due to their specific role in regulating OSCC development.

## 9 Conclusion

In summary, accumulating evidence suggests that macrophages play an increasingly pivotal role in OSCC, particularly in promoting tumor proliferation, invasion, migration, immunosuppression and immune escape. The diverse roles played by macrophages contribute to the onset and advancement of OSCC through various mechanisms. In complex immune system networks, macrophages act as sentinels, responding quickly and interacting with tumor cells and multiple immune cells. Specially, the interaction and crosstalk between OSCC cells and macrophages gradually become research hotspots. Additionally, different phenotypes of macrophages serve as important factors for evaluating treatment efficacy, prognosis, recurrence risk, and patient-specific survival rate in OSCC patients that each phenotype predicts distinct outcomes. Further elucidation of the role and mechanism of macrophages in OSCC will facilitate the discovery of effective targeted treatment strategies for OSCC involving macrophages. It also provides a detailed basis for the interaction between the immune system and tumor cells.

It is worth noting that the anti-tumoral effect of macrophages remains rare and need further research and exploration from drugs or special targeted inhibitors (17, 46, 87, 92, 94, 95, 108). Furthermore, some studies regarding the macrophages activation (87, 108) and macrophages-related prognosis (97–99) remain controversial. In terms of research method, part of the improvement needs to be tested and validated in practice. The classical polarized cell model induced by LPS and IL-4/IL-13 has a certain application market, but the tumor supernatants and tumor cells/macrophages co-culture system can better simulate the real events occurring in the OSCC tumor microenvironment, and the results are more valuable and authoritative. It is worth noting that TAMs induced by tumor supernatants or co-culture systems have their own advantages and disadvantages as well as application limitations, which can be accepted and adopted at present. Changes in the expression of pro-inflammatory and anti-inflammatory cytokines can only be used to confirm the polarization status of macrophages to a certain extent, and macrophages surface markers including CD68, CD86, and CD206 are the gold standard of polarization phenotype. Moreover, the single-cell cytokine secretion analysis platform in the engineered TME is helpful to further reveal the diverse regulation of macrophages immune response and the pro-tumoral and anti-tumoral effects of macrophages in OSCC progression (109).

Targeting macrophages holds great potential for treating OSCC. In the future researches, we would focus on the roles of TAMs-derived RNA, proteins, cytokines and metabolites in macrophages polarization. We intend to find out how RNA, proteins, cytokines and metabolites regulate the function of macrophages and how macrophages influence the OSCC progression. These strategies await further research on drugs or targeted inhibitors to make significant advancements towards reducing morbidity, mortality, and improving disease-free survival rates among OSCC patients. In addition, novel approaches targeting macrophages and other immune cells along with their interplay may hold promise as methods to impede or cure OSCC.
